# Bridging Data Gaps in Rural Oregon: A Population-Led Community Health Survey Pilot

**DOI:** 10.1093/geroni/igaf122.2765

**Published:** 2025-12-31

**Authors:** Sarah Hubner, Duyen Ngo, Jacob Melson, Russ Comer, DaiLene Wilson, Sari Hargand

**Affiliations:** Oregon Health Authority, Portland, Oregon, United States; Oregon Health Authority, Portland, Oregon, United States; Oregon Health Authority, Portland, Oregon, United States; Oregon Local Public Health Authority, John Day, Oregon, United States; Oregon Local Public Health Authority, Condon, Oregon, United States; Oregon Health Authority, Portland, Oregon, United States

## Abstract

Living in a rural area (10+ miles from a population center) is associated with increased morbidity and mortality. Rural populations are typically older, engage in fewer healthy behaviors, and have poorer access to basic resources like healthcare. Understanding the relationships between rurality, lifestyle, and health outcomes is critical to reducing disparities, particularly among older adults. Moreover, operationalizing population needs/interests supports meaningful development of actionable interventions. However, in Oregon, insufficient county-level data remains a persistent barrier to such efforts. A pilot study was therefore launched in two rural Oregon counties (Grant/Gilliam). State and local health authorities collaborated with community members to iteratively develop a comprehensive, mixed-method survey instrument (online/paper) with tailored questions on health/wellbeing, behavior, service access, and community needs. Older adults were prioritized, given Oregon’s elevated proportion of residents aged 65+, especially in rural areas (US = 17.7%, Oregon=19.6%, Grant/Gilliam=30-32%). Although data collection is ongoing (2/28/25-4/20/25, 35% complete, N = 281/800), preliminary results indicate respondents were predominantly older (55% aged 65+, range=18-85+) and female (68%). Approximately 40% reported joint/muscle/mobility issues; nearly 20% reported caregiving responsibilities. Emerging themes suggest significant barriers to healthcare, recreation, and quality produce, pointing to marked regional deficiencies. There is notable demand for local health professionals/services (primary care, dentistry, telehealth, etc.), emphasizing critical gaps in access which may compound rural health disparities. Overall, results suggest aging issues are prominent in rural Oregon, potentially exacerbating individual challenges and cyclically diminishing quality of life. This pilot can inform future development and implementation of population-led assessments to improve data availability in small/rural communities.

